# Correction: Fatty acids' double role in the prebiotic formation of a hydrophobic dipeptide

**DOI:** 10.1039/c6sc90026g

**Published:** 2016-04-19

**Authors:** Sara Murillo-Sánchez, Damien Beaufils, Juan Manuel González Mañas, Robert Pascal, Kepa Ruiz-Mirazo

**Affiliations:** a Biophysics Unit (CSIC, UPV/EHU) , University of the Basque Country , Spain . Email: kepa.ruiz-mirazo@ehu.eus; b Institut des Biomolécules Max Mousseron (IBMM, UMR 5247, CNRS/Université de Montpellier/ENSCM) , Montpellier , France . Email: robert.pascal@umontpellier.fr; c Department of Biochemistry and Molecular Biology , University of the Basque Country , Spain; d Department of Logic and Philosophy of Science , University of the Basque Country , Spain

## Abstract

Correction for ‘Fatty acids' double role in the prebiotic formation of a hydrophobic dipeptide’ by Sara Murillo-Sánchez *et al.*, *Chem. Sci.*, 2016, DOI: 10.1039/c5sc04796j.



## 

After online publication of this article (Feb 2016), a mistake in Fig. 2 was detected. All the profiles shown in the bar diagram are qualitatively correct, but the exact values (for the dodecanoic case) do not correspond to the correct values in Table S1.

A correct version of Fig. 2 is shown below:

**Fig. 2:**
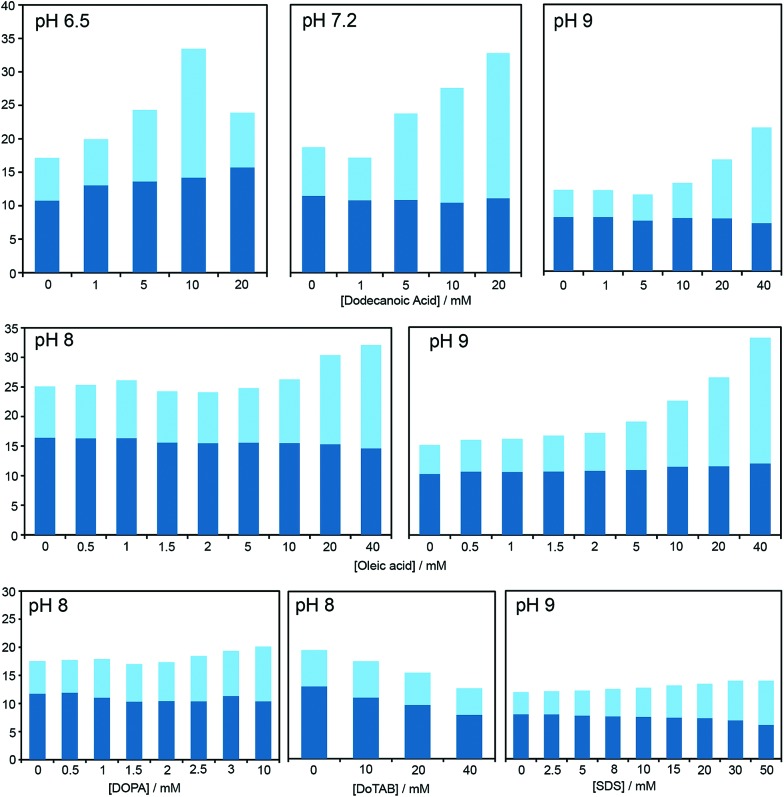
Bar graph representing the total yield of dipeptide (full bar size) and stereoselectivity (chiral conversion%: Ac-l-Tyr(Me)-l-Leu-NH_2_, in dark blue; and Ac-l-Tyr(Me)-l-Leu-NH_2_, in light blue) which are obtained in reactions carried out at room temperature, in aqueous buffer (100 mM) at various pH values, starting from Ac-Tyr(Me) oxazolone (1 mM), in the presence of H-l-Leu-NH_2_ (5 mM) and diverse amphiphile/surfactant concentrations. Each bar corresponds to a single experiment; error fluctuation was estimated with the control samples, without amphiphile (based on d.r. data and calculated for each pH value – see Table S1†) and remains within the range of 12–18% for all cases. For a more exhaustive report of the results, see main text and Table S1 in the ESI.†

The Royal Society of Chemistry apologises for these errors and any consequent inconvenience to authors and readers.

